# Low Serum Vitamin D Concentrations Are Associated with Insulin Resistance in Mexican Children and Adolescents

**DOI:** 10.3390/nu11092109

**Published:** 2019-09-05

**Authors:** Edgar Denova-Gutiérrez, Paloma Muñoz-Aguirre, Desiree López, Mario Flores, Mara Medeiros, Natalia Tamborrel, Patricia Clark

**Affiliations:** 1 Nutrition and Health Research Center, National Institute of Public Health, Cuernavaca, Morelos 62100, Mexico (E.D.-G.) (M.F.); 2 CONACYT-Center for Population Health Research, National Institute of Public Health, Cuernavaca, Morelos 62100, Mexico; 3 Clinical Epidemiology Research Unit, Hospital Infantil de México Federico Gómez, Mexico City 06720, Mexico (D.L.) (P.C.); 4 Nephrology and Bone Mineral Metabolism Research Unit, Hospital Infantil de México Federico Gómez, Mexico City 06720, Mexico; 5 School of Medicine, Universidad Nacional Autónoma de México, Mexico City 04510, Mexico

**Keywords:** vitamin D, insulin resistance, diabetes, children, adolescents

## Abstract

Previous studies in the Mexican adult population have suggested a relationship between low levels of serum concentrations of serum vitamin D with impaired glucose tolerance, metabolic syndrome, and diabetes, regardless of the presence of obesity. The aim of this study is to investigate the relationship between serum vitamin D levels and the factors linked to insulin resistance. A total of 533 children and adolescents from the “Reference Values of Body Composition in the Pediatric Population of Mexico City” study are assessed. Body composition, dietary, and lifestyle data are obtained. Serum vitamin D, insulin, and glucose are also measured. Associations are tested using multiple linear and logistic regression models. Approximately 90% of children and adolescents in this study have sub-optimal vitamin D levels (<30 ng/mL). An inverse relationship between insulin resistance and serum vitamin D is observed (OR (odds ratios) = 2.9; 95% CI (95% confidence intervals): 1.1, 7.2; *p-*trend 0.03). Low serum vitamin D levels are associated with insulin resistance in the pediatric population. The present study provides additional evidence for the role of vitamin D in insulin resistance. Our findings suggest the supplementation of vitamin D may be helpful in preventing insulin resistance and subsequent diabetes.

## 1. Introduction

Diabetes is one of the leading causes of morbidity and mortality globally [1]. Defects in pancreatic β-cell function, insulin sensitivity, and systemic inflammation all contribute to the development of diabetes [2]. Insulin resistance (IR) is described as the inadequate response of the skeletal muscle, liver, and adipose tissue to the endogenous insulin secretions. In addition to β-cell dysfunction, IR plays an important role in the pathogenesis of diabetes [3]. Since IR is a risk factor for diabetes, understanding the role of various nutritional and other modifiable risk factors that may contribute to the pathogenesis of diabetes is essential [2]. In the Mexican adult population, the joint prevalence of 25(OH)D (vitamin D) deficiency and insufficiency is 11.8% [4]. Globally, serum vitamin D deficiency is a major public health problem in all age groups, even in populations residing in countries near the equator, where it was generally assumed that sun exposure was adequate enough to prevent this deficiency, and in industrialized countries, where vitamin D fortification has been implemented for years [5].

Previous studies in the Mexican adult population have suggested a relationship between low levels of serum concentrations of serum vitamin D with impaired glucose tolerance, metabolic syndrome, and diabetes, regardless of the presence of obesity [6,7,8,9,10]. Using the method considered the gold standard (the hyperglycemic clamp), levels of serum vitamin D were positively associated with insulin sensitivity (IS), and negatively associated with the secretion of first and second phase insulin. Chiu et al. reported subjects with serum vitamin D deficiency (<20 ng/mL) had an increased risk of IR and metabolic syndrome [11]. Additionally, in a recent study, also with the Mexican adult population, a significant inverse correlation was found between serum vitamin D concentrations and the risk of diabetes, as well as subclinical inflammation [12].

Childhood and adolescence are important stages of growth and development in which multiple processes of change and adaptation can be related to critical health conditions. In Mexico, pediatric overweight and obesity are a major public health problem [13], and this phenomenon is also linked to other metabolic disorders and comorbidities as an adult, such as IR and diabetes [14,15]; therefore, the analysis of factors related to these two conditions, such as serum vitamin D concentrations, is preponderant.

Although some studies suggest that vitamin D deficiency is a risk factor associated with altered fasting glucose values and IR in humans, it remains a controversial issue, especially in children. In addition, short-term supplementation studies have produced conflicting results on the effect of vitamin D on glucose tolerance and IR; thus, the present study attempts to evaluate the association between serum vitamin D concentrations and IR in children and adolescents from Mexico City.

## 2. Methods

### 2.1. Study Design and Population

Data were used from the study “Reference Values of Body Composition in the Pediatric Population of Mexico City”, which is a representative sample from schools in Mexico City of apparently healthy children and adolescents ranging in age from 5–20 years. The direct indicators of wellness and health were measured among this population.

The original study included children and adolescents with availability to attend the hospital for periodic assessment. Children who presented chronic-degenerative diseases, endocrinological diseases, systemic diseases, respiratory diseases, neurological diseases, cardiological diseases, heart failure, renal failure, psychiatric disorders, chromosomopathies, genopathies, dysmorphic syndromes, and systemic arterial hypertension were excluded from the study. Subjects who were receiving systemic pharmacological treatment, which affects lipid metabolism or glucose, and adolescents with current or previous gestation were also excluded from the study.

The present study was executed according to the Helsinki Declaration guidelines. The Research, Ethics, and Biosecurity Committee at INSP reviewed and approved the study protocol and informed consent forms. Written informed consent forms were obtained from each participant and their parents.

### 2.2. Demographic and Lifestyle Measures

The demographic characteristics were obtained from questionnaires applied by trained personnel. Physical activity was estimated using a physical activity questionnaire (International Physical Activity Questionnaire: IPAQ-short) adapted and validated for the Mexican population, in which participants were asked about their daily recreational activity. Additionally, information was obtained on the physical activity of free time in hours/day, as well as minutes/day.

Pubertal development of the participants was evaluated according to Tanner’s theoretical scale. These data were obtained in medical examination by pediatrics.

Diet was assessed using a previously validated 116 items semi-quantitative food frequency questionnaire [16]. For each item, participants were asked to specify how often, on average, over the previous year they consumed the food or beverage. The frequency of consumption responses was categorized as never, <1 a month, 1–3 times a month, once a week, 2–4 times a week, 5–6 times a week, once a day, 2–3 times per day, 4–5 times per day, and ≥6 times per day. Energy and nutrient intake were calculated by multiplying the frequency of consumption of each unit of food by the energy and nutrient content of the specified portion size. A Mexican database of food contents was used to assess the composition values for energy and nutrients [17].

### 2.3. Biologic and Anthropometric Measures

Anthropometric information was obtained by trained personnel using standardized procedures. The participants were weighed on a previously calibrated electronic scale (SECA) with minimal clothing and without shoes. The size was evaluated using a stadiometer of the same brand. Normal weight was defined as body mass index (BMI) WHO’s references [18]: overweight: >+1SD (equivalent to BMI 25 kg/m^2^ at 19 years), obesity: >+2SD (equivalent to BMI 30 kg/m^2^ at 19 years), and thinness: <−2SD.

Body fat ratio was evaluated by dual energy, X-ray absorption (DXA) (GE Healthcare, version 15, Chicago, IL, USA).

### 2.4. Insulin Resistance and Vitamin D

Blood samples were obtained after a fasting time of at least 8 h. Serum glucose levels were evaluated by the oxidized glucose method, and insulin was determined by direct radioimmunoassay method in solid phase. Serum concentrations of vitamin D were measured through chemiluminescence assay by using the Liaison 25-hydroxyvitamin D total assay (DiaSorin Inc., Stillwater, MN, USA). Moreover, based on preliminary testing using external quality controls from Bio-Rad (Bio-Rad Laboratories Inc., Hercules, CA, USA) and DiaSorin, the within-run and between-run coefficients of variation for this assay varied from 3.2–8.5% and 6.9–12.7%, respectively.

To determine vitamin D deficiency and insufficiency, the following cut-off points were used [15]: severe deficiency defined as <8 ng/mL (<20 nmol/L), moderate deficiency as 8–20 ng/mL (20–50 nmol/L), insufficiency as 20–30 ng/mL (50–75 nmol/L), and adequacy as ≥30 ng/mL (≥75 nmol/L).

Homeostasis model assessment index (HOMA index) was used to assess IR, and it was calculated from fasting insulin and glucose, using the following formula: fasting insulin concentration (U/L) X fasting glucose concentration (mg/dL)/405. Finally, subjects with HOMA index ≥3.16 were diagnosed as insulin-resistant. This cut-off point has been used in similar populations in Latin America and is similar to that proposed by Keskin et al. in the pediatric population [19].

### 2.5. Statistical Analysis

All statistical analyses were performed in STATA 13.0. The Kolmogorov–Smirnov test was used to evaluate the normality of variables, performing a logarithmic transformation in those variables that did not present a normal distribution. A descriptive analysis of the main variables of interest was carried out.

Additionally, the distribution of the variables of interest was evaluated according to the tertile of vitamin D. One-way ANOVA was used to test for a linear trend across tertiles of plasma vitamin D concentrations, while, chi-square tests were used to evaluate the distribution of qualitative variables across vitamin D tertiles. In addition, to assess the association between HOMA index and serum vitamin D concentrations, multiple linear regression models were computed. Finally, to assess the association between IR and serum vitamin D concentrations, odds ratios (OR) and 95% confidence intervals (95% CI) were calculated using multivariate logistic regression models.

## 3. Results

For the present study, a total of 533 children and adolescents were included. Of these, 45.8% were girls and 54.2% were boys. The mean age of the study population was 11.6 years. Clinical and anthropometric characteristics of the study population showed that 54.3% of the sample was concentrated in stages of Tanner I and II. According to the body mass index, 22.1% were overweight and 9.0% were obese. For girls, mean of HOMA index of all subjects was 1.9 ± 1.4. According to the HOMA-IR index, 9.9% of the study subjects showed IR (10.3 vs. 9.7 for girls and boys, respectively). We found that vitamin D concentrations of 90% of all subjects were below the normal threshold (<30 ng/mL). Of these, 42.9% of all studied subjects were vitamin D-deficient (<20 ng/mL) (Table 1).

Serum vitamin D concentrations were divided into tertiles. In this sense, for tertile 1 (low) the mean serum vitamin D was 15.1 ng/mL, tertile 2 (medium) had a mean of 21.3 ng/mL, and for tertile 3 (high) a mean serum vitamin D of 29.2 ng/mL. Approximately 90% of children and adolescents in this study had sub-optimal vitamin D levels.

Fat mass across tertiles of vitamin D was 28.9%, 31.1%, and 33.0% (for high, medium, and low tertiles, respectively) (Table 2).

**Figure 1:**
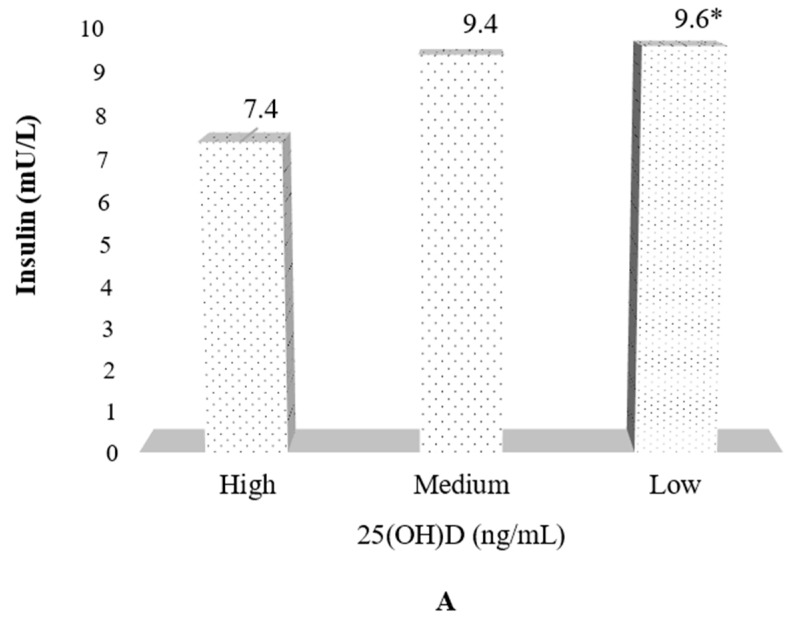

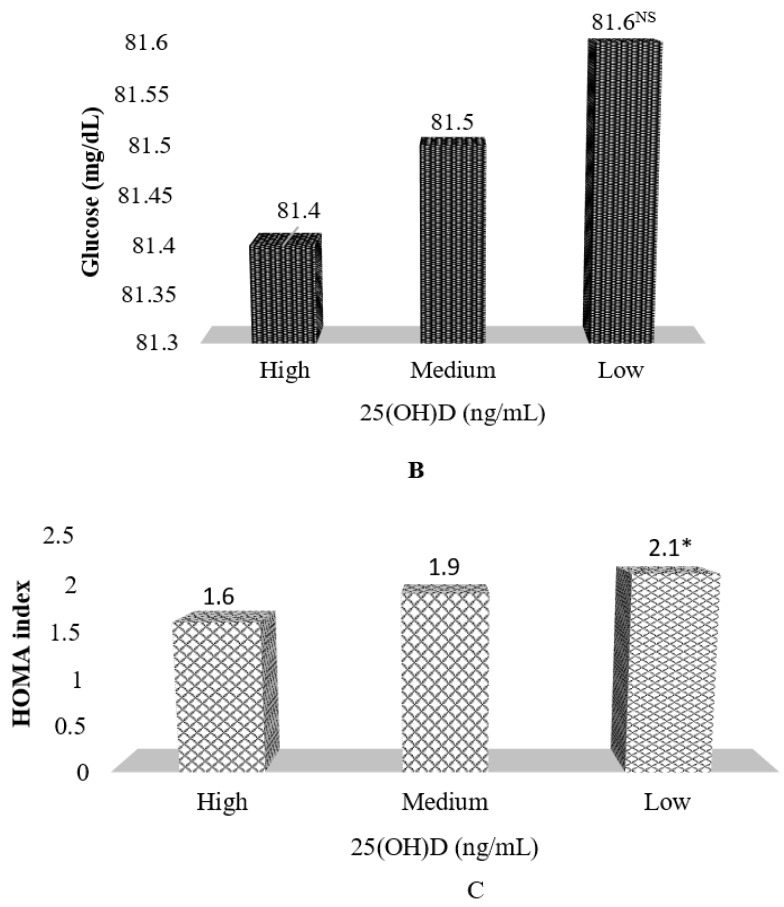
(**A**–**C**) HOMA index and related markers ^1^ by tertile of vitamin D levels. (**A**) Insulin levels by tertile of serum vitamin D; (**B**) Glucose levels by tertile of serum vitamin D; (**C**) HOMA index ^δ^ by tertile of serum vitamin D. ^1^ Values are adjusted by age, sex, BMI, Tanner stage. ^δ^ Homeostasis Model Assessment (HOMA index) was calculated as fasting insulin (U/L) × fasting glucose (mg/dL)/405. * *p* < 0.001; NS: not statistically significant.

After adjusting for age, sex, BMI, and Tanner stage, with respect to the insulin levels, mean values of 9.6, 9.4, and 7.4 were observed in tertiles low, medium, and high, respectively. Additionally, for the HOMA-IR index, mean values of 2.1, 1.9, and 1.6 were observed in tertiles low, medium, and high, respectively (Figure 1A–C).

The multivariate linear regression relationship between vitamin D concentrations and HOMA index in Mexican children and adolescents is presented in Table 3. Our data suggest that HOMA-IR index and insulin levels were inversely associated with vitamin D concentrations. Additionally, when we evaluated the association between serum vitamin D concentrations and the presence of IR in children and adolescents from Mexico City, and after adjusting for age, sex, BMI, Tanner stage, physical activity, and energy intake, we observed that subjects in the lowest tertile of serum concentrations of vitamin D were likely to have IR (OR = 2.9; 95% CI: 1.1, 7.2; *p*-trend 0.030) when compared to subjects in the highest tertile of vitamin D (Table 4). Finally, we evaluated the association between serum vitamin D concentrations and the presence of IR in children and adolescents from Mexico City using predefined cut-offs for vitamin D deficiency and insufficiency. In this case, we observed that subjects with deficient concentrations of vitamin D (<20 ng/mL) have two-fold higher odds of IR (OR = 2.1; 95% CI: 1.0, 9.5; *p*-trend 0.055) (Supplementary Table S1); however, this association was marginally significant.

## 4. Discussion

To summarize, in children and adolescents from Mexico City, low serum vitamin D levels were associated with higher odds of insulin resistance. With respect to the prevalence of overweight and obesity, our data suggest a joint prevalence of 32.8% (24.6% of overweight and 8.2% of obesity). This finding is similar to the data shown by Mexican National Health and Nutrition Survey (ENSANUT, by its Spanish acronym) 2016, which reported a combined prevalence of overweight and obesity of 33.2% in subjects of school age, and of 36.3% in adolescents [20].

Vitamin D deficiency in Mexico is an important public health problem. In our study, approximately 90% of all studied subjects had suboptimal vitamin D levels (<30 ng/mL), and 42.9% of all studied subjects were vitamin D-deficient (<20 ng/mL), which was higher than the reported rate from the ENSANUT-2006 and similar to a recent study in Mexican children in which 43.6% of the sample had hypovitaminosis D [21,22]. ENSANUT-2006 reported 54% vitamin D deficiency and insufficiency in preschool children and 28% scholars, while in adolescents, the joint prevalence was 30.1% [21]. In 2008, a multicenter study conducted in children between 3 and 8 years old, vitamin D deficiency was reported in 25% of the subjects, while the frequency of subjects with vitamin D insufficiency was 63%, meaning only 12% of the population had optimal vitamin D levels. In a more recent study conducted in 2015, which included a sample of 261 children and adolescents for measurement of serum vitamin D, it was found that 60.9% fell into the category of insufficient, and only 29.1% were classified as sufficient, according to the references used [23].

According to our findings, a negative association is observed between serum vitamin D and overweight and obesity. Our data suggest that the prevalence of overweight/obesity in the study population was lower when the serum concentrations of vitamin D were higher: 22.0% in the highest tertile of serum vitamin D vs. 36.8% in the lowest tertile of serum vitamin D. In this sense, our data are consistent with those reported by Contreras-Manzano et al., who suggested that subjects with insufficient/deficient vitamin D concentrations are more likely to be overweight/obese, although these results are observed in the Mexican adult population [24]. Similarly, our findings suggest that there is a lower percentage of fat mass as the concentration of serum VD increases, with frequencies of 32.8%, 31.4%, and 29.2% for tertiles 1, 2, and 3, respectively; this has also been observed in prepubertal Chilean children [25]—Cediel et al. found serum vitamin D was inversely associated with indicators of total and central adiposity and with IR indicators. Effect sizes were moderate in girls (~0.3 for adiposity and IR indicators), while weaker values were found in boys.

As mentioned above, we found a relationship between vitamin D and IR; in the present work, a higher odd of presenting IR (OR = 2.9; 95% CI: 1.1, 7.2, *p*-trend = 0.03) was observed in the population with lower levels of serum VD. These findings are consistent with what has been described in other populations. For example, in a study conducted in Korea (subjects between 10 and 19 years), authors stratified participants into three serum vitamin D categories (<37.5 nmol/L, 37.5 to <50 nmol/L, and ≥50 nmol/L), and significantly decreasing trends were observed for fasting insulin (all *p* < 0.001) and HOMA-IR (all *p* < 0.001) [26]. Similarly, in Chile, Cediel et al. found an increased risk of developing hyperinsulinism and IR in subjects with deficient serum vitamin D concentrations (OR = 2.9 (95% CI 1.2, 7.1), OR = 3.3 (95% CI 1.6, 7) in girls and boys, respectively), compared to those subjects who had sufficient levels of serum vitamin D [25].

The present study has limitations due to its design and scope. First, because of its cross-sectional design, it is not possible to infer a causal relationship between serum vitamin D concentrations and IR. Despite having observed a significant association between serum vitamin D and the prevalence of IR in children and adolescents from Mexico City, our sample size was small, and therefore this did not allow us to perform a stratified analysis by some variables of interest as the BMI. The high prevalence of one of the variables of interest caused little variability in the data, biasing the results downwards. In this sense, for the present work, serum concentrations of vitamin D were stratified in tertiles, which made our groups incomparable with other studies. The little variability between the data made it difficult to establish the initial hypothesis of sufficiency as a protective factor and deficiency as a risk factor. However, we observed that as the serum concentrations of vitamin D increased, the possibility of presenting IR decreased. On the other hand, in terms of external validity, due to the characteristics of the study population, our results cannot be extrapolated to the whole country or to other age groups. Additionally, measurements of serum vitamin D were performed using a method that is not considered as the gold standard. However, previous studies have shown good correlation (*r* = 0.88) between the method used in the present work and the gold standard for measuring serum vitamin D [27].

## 5. Conclusions

Low serum vitamin D levels are associated with insulin resistance in the pediatric population. Most children and adolescents in this study had sub-optimal vitamin D levels, and the cause of the deficiency may be due to different factors mentioned previously. Flores et al. mentioned that school-age children consume a quarter (from 90–120 IU of vitamin D3) of the 400 IU recommended by the Institute of Medicine of the United States for the pediatric population [28]. On the other hand, scarce solar exposure and the existence of sun protection methods, as well as aforementioned physical, clinical, and environmental factors, may be related to low vitamin D concentrations in the Mexican pediatric population. If causally associated, the supplementation of vitamin D may help in preventing insulin resistance and subsequent diabetes.

## Figures and Tables

**Table 1:** Characteristics of the study population, by gender.

Variable	Girls (*n* = 244)	Boys (*n* = 289)	Total (*n* = 533)
Age ^1^, years	11.7 ± 4.1	11.5 ± 3.5	11.6 ± 3.9
Weight, kg	42.4 ± 17.4	44.4 ± 17.2	43.5 ± 17.3
Height, cm	142.2 ± 16.6	147.5 ± 19.4 ***	145.1 ± 18.4
BMI ^Φ^, kg/m^2^			
Normal, %	170 (69.6)	198 (68.4)	367 (68.9)
Overweight, %	57 (23.4)	60 (20.8)	118 (22.1)
Obesity, %	17 (7.0)	31 (10.8)	48 (9.0)
Body fat percentage,	33.9 ± 6.9	28.5 ± 8.6 ***	31.2
Triglycerides, mg/dL	92.7 ± 10.7	89.2 ± 6.8	90.9 ± 8.8
HDL-c ^ϕ^, mg/dL	55.2 ± 13.2	55.6 ± 13.7	55.4 ± 13.6
Glucose, mg/dL	80.4 ± 7.7	82.3 ± 7.1 ***	81.4 ± 7.5
Insulin, mU/L	9.9 ± 6.6	8.4 ± 5.2 **	9.1 ± 5.9
HOMA index ^δ^	1.9 ± 1.4	1.7 ± 1.1 ***	1.8 ± 1.3
Insulin resistance ^Ω^, %	25 (10.3)	28 (9.7) *	53 (9.9)
Tanner, %			
I	93 (37.9)	129 (44.6)	221 (41.5)
II	32 (13.3)	36 (12.3)	68 (12.8)
III	28 (11.7)	36 (12.6)	65 (12.2)
IV	47 (19.2)	56 (19.3)	103 (19.2)
V	44 (17.9)	32 (11.2)	76 (14.3)
25(OH)D, ng/mL	20.4 ± 6.3	22.8 ± 6.4 ***	21.7 ± 6.5
≥30, %	20 (8.2)	38 (13.2) ***	58 (10.9)
≥20 and <30, %	99 (40.6)	147 (50.9) *	246 (46.2)
<20, %	125 (51.2)	104 (35.9) ***	229 (42.9)
Energy intake, kcal/day	2390.0 ± 1069.4	2611.9 ± 1056.1 *	2496.8 ± 1068.2
Physical activity, min/day	39.2 ± 38.7	58.0 ± 57.6 **	48.2 ± 49.6

^1^ Values are mean ± SD and *n* (%) per group for all other variables; ^Φ^ body mass index (BMI: kg/m^2^); ^ϕ^ high-density lipoprotein (HDL-c); ^δ^ homeostasis model assessment (HOMA index) was calculated as fasting insulin (mU/L) × fasting glucose (mg/dL)/405; ^Ω^ Cut-off point for diagnosis of insulin resistance is 3.16. * *p* < 0.05, ** *p* < 0.01, and *** *p* < 0.001.

**Table 2:** Characteristics of the study population, by vitamin D tertile ^1^.

Variable	High *n* = 173	Medium *n* = 175	Low *n* = 183
25(OH)D (ng/mL)	29.2 ± 3.9	21.3 ± 1.6	15.1 ± 2.6 ***
Age (years)	10.7 ± 3.9	11.7 ± 3.7	12.4 ± 3.7
Women	59 (34.1)	81 (46.3)	103 (56.3) ***
Weight (kg)	38.2 ± 16.4	45.1 ± 17.8	46.9 ± 16.5 **
Height (cm)	140.8 ± 20.1	146.2 ± 18.5	148.1 ± 15.8 ***
BMI ^Φ^ (kg/m^2^)			
Overweight	28 (16.2)	40 (22.9)	49 (26.8) **
Obesity	10 (5.8)	21 (12.0)	18 (10.0) **
Body fat	28.9 ± 7.7	31.1 ± 8.3	33.0 ± 8.2 **
Glucose (mg/dL)	81.4 ± 7.5	81.8 ± 7.3	81.5 ± 7.5
Insulin (mU/L)	7.0 ± 3.7	9.5 ± 5.8	10.7 ± 7.1 **
HOMA index ^δ^	1.4 ± 0.8	1.9 ± 1.2	2.2 ± 1.6 ***
Insulin resistance, (%) ^Ω^	4.0	11.4	13.8 ***
Tanner, (%)			
I	90 (52.3)	66 (37.6)	64 (34.8) **
II	18 (10.5)	26 (15.0)	23 (12.4)
III	13 (7.5)	26 (15.0)	26 (14.0)
IV	27 (15.7)	29.4 (16.8)	46 (25.3)
V	24 (14.0)	27 (15.6)	25 (13.5)

^1^ Values are mean ± SD and *n* (%) per group for all other variables; ^Φ^ BMI: kg/m^2^; ^δ^ HOMA index was calculated as fasting insulin (mU/L) × fasting glucose (mg/dL)/405; ^Ω^ cut-off point for diagnosis of insulin resistance is 3.16. * *p* < 0.05, ** *p* < 0.01, and *** *p* < 0.001.

**Table 3:** Multivariate linear regression analysis to assess relationship between vitamin D concentrations and HOMA index, glucose, and insulin in Mexican children and adolescents.

Variable	Crude			Adjusted ^		
	β	SE	*p*-Value	β	SE	*p*-Value
Glucose (mg/dL)	−0.11	−0.210	0.409	−0.02	0.051	0.72
Insulin (mU/L)	−0.24	0.030	<0.001	−0.14	0.033	<0.001
HOMA index ^δ^	−0.05	0.008	<0.001	−0.03	0.007	<0.001

^ Adjusted by age (years), sex, BMI (normal, overweight, obesity), Tanner stage, physical activity (min/day), and energy intake (kcal/day); ^δ^ HOMA index was calculated as fasting insulin (U/L) × fasting glucose (mg/dL)/405.

**Table 4:** Odds ratio of the association between vitamin D concentrations and insulin resistance in Mexican children and adolescents.

Variable	Crude			Adjusted ^		
	OR	95% CI	*p* Trend	OR	95% CI	*p* Trend
Vitamin D tertile						
Medium	3.0	1.3, 7.4	0.003	2.0	0.8, 5.2	0.030
Low	3.8	1.6, 8.9		2.9	1.1, 7.2	

^ Adjusted by age (years), sex, BMI (normal, overweight, obesity), Tanner stage, physical activity (min/day), and energy intake (kcal/day).

## Supplementary Materials

The following are available online at https://www.mdpi.com/2072-6643/11/9/2109/s1, Table S1. Odds Ratio of the association between suboptimal vitamin D concentrations and insulin resistance^1^ in Mexican children and adolescents.
