# Supplementary material for: Low Serum Vitamin D Concentrations Are Associated with Insulin Resistance in Mexican Children and Adolescents
Source: Nutrients. 2019 Sep 5;11(9):2109. doi: 10.3390/nu11092109 (PMC6770751; doi:10.3390/nu11092109)
Supplement: Supplementary file 1 [file nutrients-11-02109-s001.pdf]

Supplementary Material

Supplementary table 1. Odds Ratio of the association between suboptimal vitamin D concentrations and insulin resistance[1] in Mexican children and adolescents.

Variable	Crude			Adjusted*		
	**OR**	**95% CI**	**P trend**	**OR**	**95% CI**	**P trend**
**Vitamin D cutoffs**						
**≥ 20-<30 ng/mL**	2.5	0.6, 7.9	0.010	1.3	0.5, 6.2	0.055
**< 20 ng/mL**	4.4	1.7, 9.8		2.1	1.0, 9.5	

*Adjusted by age (years), sex, BMI [normal, overweight, obesity), Tanner stage, physical activity (min/day), energy intake (kcal/day).
[1] Cutoff point for diagnosis of insulin resistance was 3.16.
